# A Multi-Step miRNA-mRNA Regulatory Network Construction Approach Identifies Gene Signatures Associated with Endometrioid Endometrial Carcinoma

**DOI:** 10.3390/genes7060026

**Published:** 2016-06-02

**Authors:** Hanzhen Xiong, Qiulian Li, Ruichao Chen, Shaoyan Liu, Qiongyan Lin, Zhongtang Xiong, Qingping Jiang, Linlang Guo

**Affiliations:** 1 Department of Pathology, Zhujiang Hospital, Southern Medical University, Guangzhou 510515, China; xionghanzhen@126.com; 2 Department of Pathology, the Third Affiliated Hospital, Guangzhou Medical University, Guangzhou 510150, China; qiulianli12@sina.com (Q.L.); ruichaochen1@sina.com (R.C.); shaoyanliu2009@sina.com (S.L.); zhongtangxiong2008@163.com (Z.X.); jiangqp2003@126.com (Q.J.); 3 Department of Gynaecology, The Third Affiliated Hospital, Guangzhou Medical University, Guangzhou 510150, China; wotooqiwawa@163.com

**Keywords:** CPEB1, endometrial carcinoma, miRNA targets, protein-protein interaction network, EMT, p53

## Abstract

We aimed to identify endometrioid endometrial carcinoma (EEC)-related gene signatures using a multi-step miRNA-mRNA regulatory network construction approach. Pathway analysis showed that 61 genes were enriched on many carcinoma-related pathways. Among the 14 highest scoring gene signatures, six genes had been previously shown to be endometrial carcinoma. By qRT-PCR and next generation sequencing, we found that a gene signature (CPEB1) was significantly down-regulated in EEC tissues, which may be caused by hsa-miR-183-5p up-regulation. In addition, our literature surveys suggested that CPEB1 may play an important role in EEC pathogenesis by regulating the EMT/p53 pathway. The miRNA-mRNA network is worthy of further investigation with respect to the regulatory mechanisms of miRNAs in EEC. CPEB1 appeared to be a tumor suppressor in EEC. Our results provided valuable guidance for the functional study at the cellular level, as well as the EEC mouse models.

## 1. Introduction

MicroRNAs (miRNAs) are a family of small (~22 nucleotides), non-coding RNA molecules that have been found in plants, animals, and some viruses. MicroRNAs regulate gene expression at the post-transcriptional level by binding to the complementary sequences of target mRNAs at the 3′UTR, and are associated with a variety of pathologic conditions, including solid and hematologic malignancies. MicroRNAs, such as hsa-miR-503, hsa-miR-205, and hsa-miR-200b, have been shown to be dysregulated in endometrioid endometrial carcinoma (EEC) [1,2].

EEC is the most dominant subtype of endometrial cancer [3]; however, knowledge regarding the molecular mechanism underlying the etiology of EEC is limited. Aberrant transcription levels of miRNAs have been observed in EEC, and cumulative evidence indicates that miRNAs may act as oncogenes or tumor suppressors in the tumorigenesis and progressing of EEC [2,4,5]. Indeed, as many as 118 differentially expressed miRNAs have been reported by 41 endometrial cancer publications. Therefore, it is critical to identify the clinically important miRNAs that contribute to endometrial cancer progression and further understand the miRNA regulatory mechanism underlying endometrial cancer. To that end, we have developed the miRNA-mRNA interaction network that describes the endometrial cancer regulatory mechanism. 

In our previous research [6], we identified five differentially expressed miRNAs and 10 target genes in stage I EEC tissues by next generation sequencing, and constructed a miRNA-gene regulatory network related to cell cycle pathway. The miRNA-gene regulatory network has been reported in some diseases, such as lung cancer [7], nasopharyngeal carcinoma [8], and prostate cancer [9]. The networks in those publications were constructed based on the correlation between the level of miRNA and mRNA expression. From those miRNA-gene networks, we showed that one miRNA does not just mediate one gene, but one or many miRNAs mediate one or many genes at the same time. For example, hsa-miR-15a, hsa-miR-26a, and hsa-miR-29c regulated HDAC1, RB1, SMARCA5, TBL1XR1, and XRCC6 in prostate cancer [9]. Moreover, the generally accepted criteria for miRNA target prediction are the sequence complementarities between the miRNA seed region (2′8 nucleotides at the 5’end) and the target mRNAs. This would commonly give rise to hundreds of target candidates for one given miRNA. Thus, beginning with those 118 miRNAs, we tried to combine a traditional miRNA target prediction method and protein-protein interaction analysis method for searching a potential regulatory network. Those genes in the network are gene signatures related to endometrial carcinoma. The method we developed to identify miRNA target genes is superior to the traditional one-to-one method. Our method of searching for gene signatures can be applied to any known disease-related miRNA pools. 

## 2. Results

We presented a pipeline for identifying gene signatures by constructing miRNA-mRNA regulatory networks specific to endometrial carcinoma. For identifying gene signatures by constructing miRNA-mRNA regulatory networks specific to endometrial carcinoma, our multi-step bioinformatics approach was developed based on the traditional target prediction by miRNA seed-theory, and provided a novel genome-scale overview of the miRNA-mRNA interaction and PPI network in EEC. 

### 2.1. Endometrial Carcinoma-Related miRNAs

Based on the four databases, we identified a total of 118 miRNAs; however, those curated databases included in our analysis were constructed using different methods. PhenomiR and hmdd are databases constructed based on multiple bioinformatics analysis, combined with either numerous miRNA expression experiments or simply the seed math algorithm. miRwalk is a database curated by Pubmed text mining, and the experimentally validated miRNAs in endometrial carcinoma can only be extracted from the miR2disease database. We chose the miRNA-disease relationships that were reported in at least two of the four databases or documented in no less than two independent studies to ensure that the EEC relevance of our miRNA list was verified by a different bioinformatics algorithm or verified by both bioinformatics algorithms and experimental evidence. As a result, we obtained reliable endometrial carcinoma-related miRNA containing 35 miRNAs. 

### 2.2. Initial Prediction of Endometrial Carcinoma Related Genes

We obtained 110,995 miRNA-mRNA interactions (data not shown) by compiling a non-redundant list of predicted targets using the four miRNA-target prediction algorithms described in the Materials and Methods section. MicroRNA-mRNA pairs supported by less than two algorithms were eliminated, and the number of miRNA-mRNA pairs decreased to 7919, consisting of 27 miRNAs and 3082 target genes. Four hundred fifty-seven genes that were associated with ≥5 of the 27 miRNAs were considered to be the initial high-confidence targets.

### 2.3. Protein-Protein Interaction Analysis Identified High-Confidence Endometrial Carcinoma-Related Genes

Protein–protein interactions (PPIs) refer to intentional physical contacts between two or more protein molecules due to biochemical events and/or electrostatic forces. Protein-protein interaction analysis was performed on the 457 initial candidates, and we found that most of protein-protein interactions were closely related (Figure S1). We calculated scores of these 457 candidates and ranked them according to the score in descending order. We found that 61 of the potential targets possess a high score (≥5). These 61 genes were then classified as high-confidence endometrial carcinoma-related genes (Figure S2). 

### 2.4. Evaluation of 61 High-Confidence Endometrial Carcinoma-Related Genes

As the preliminary verification of function, a biological pathway analysis was conducted to determine the statistically-enriched pathways among the top 61 genes (Table 1). A number of pathways appeared to be carcinoma-related, including pathways involved in bladder, prostate, and endometrial carcinomas. We combined the interactions of 61 genes and the relationships with miRNAs, which were visualized by Cytoscape software (Figure S2). To validate the reliability of our prediction, we performed a literature research on these initial endometrial carcinoma-related genes. Interestingly, we found that 6 of the top 14 genes (genes with >10 neighbor genes) have been well-studied for involvement in endometrial carcinoma. In 1997 Tashiro [10] proved that PTEN is a tumor suppressor gene, indicating that mutations in PTEN play a significant role in the pathogenesis of EEC. Early in 1998, Risinger *et al*. [11] found that PTEN mutations are associated with endometrioid histology and other favorable pathologic, clinical, and molecular features rather than with increased metastatic potential, as has been noted in other types of cancers [11]. Other gene interactions are shown in Table 2.

### 2.5. Validation of Interesting Endometrial Carcinoma-Related Genes

In order to verify the differential expression of our predicted targets in EEC, qRT-PCR was performed. Ten genes were selected for expression analysis in six carcinoma and matched adjacent normal tissues (Table 3). We found that three of the genes were significantly dysregulated in endometrial carcinoma tissues compared to normal endometrium. Two genes (CDC25A and IGF1R) were up-regulated (*p* = 0.04) in endometrial carcinoma, and CPEB1 was down-regulated (*p* = 0.05). Then, we checked the level of expression of the three genes in our RNA sequencing experiments on three pairs of stage I EEC tissues, and found that only the CPEB1 gene was significantly different (*p* = 0.0003). We chose CPEB1 for large-scale validation in 16 pairs of EECs and corresponding normal tissues, and showed that CPEB1 expression was significantly suppressed in EEC (*p* = 0.003). 

### 2.6. CPEB1 May Be Relevant to up-Regulated hsa-miR-183-5p in EEC Tissues

Because CPEB1 may play an important role in EEC, we surveyed the potential up-stream miRNAs of CPEB1. CPEB1 has four groups of potential up-stream miRNAs predicted by our prediction methods described in the Materials and Methods section, including hsa-let-7, hsa-miR-129-2-3p, hsa-miR-183-5p, and hsa-miR-96-5p. We sequenced all miRNAs on three pairs of stage I EEC tissue samples. After we calculated the RPKM value for the four groups of miRNAs, we found that the level of hsa-miR-183-5p expression was significantly higher in EEC samples than corresponding normal samples (*p* = 0.0458). It is noteworthy that hsa-miR-183-5p is predicted to bind to position 454–460 of CPEB1 3′-UTR by Targetscan software.

## 3. Discussion

Endometrial carcinomas are the most frequent carcinoma occurring in the female genital tract (http://seer.cancer.gov/), and EEC is the most dominant subtype [29]. Although stage I EEC patients have a lower mortality rate than other stage types of EEC after an aggressive surgical approach, those surgeries, such as removing the uterus and cervix, ovaries, and fallopian tubes, render patients with a strong desire to be pregnant infertile. Our pathway analysis showed that the 61 initial EEC-related gene set has a great enrichment probability (correlation *p* value = 0.0000947) in the “Endometrial Carcinoma” pathway (KEGG: 05213). The major pathways associated with endometrial carcinoma based on our biological pathway analysis included signal transduction, cell cycle, prostate carcinoma, or other carcinoma-related pathways, immune system, and oocyte meiosis. In 2014 Wang *et al.* [30] reported that endometrial tumor growth is promoted by cell cycle acceleration. These results were consistent with our prediction. Moreover, our pathway analysis showed that endometrial carcinoma-related genes were commonly involved in certain pathways related to the reproductive system, such as the prostate carcinoma and oocyte meiosis pathways. Early this year, it was found that uterine and prostate carcinomas might be linked to aberrant transcription according to a genome-wide association study [31], which was consistent with our conclusions. The enriched pathways also supported the reliability of our multi-step prediction approach.

From the qRT-PCR assay with seven pairs of EECs and adjacent normal endometrium tissues, 3 of the 10 genes examined were significantly dysregulated in ECC tissues. CDC25A was shown to be up-regulated 27.322-fold in ECC tissues. Our results are consistent with the scientific report from Patel *et al.* [22], which showed that CDC25 family expression is up-regulated in various types of cancers, including endometrial carcinoma. Increased transcription of insulin-like growth factor-I receptor (IGF1R) gene has been reported, and was confirmed in our EEC patients (4.742-fold). The CPEB1 gene was down-regulated by 0.417-fold in EEC patients, even though involvement of CPEB1 in EEC has never been demonstrated. Down-regulation of CPEB1 was also found in our previous results of a next generation sequencing project with three EEC patients (NIH Short Read Archive database SRP045645).

It has been reported that hsa-miR-183-5p is highly expressed in HEC-1B cells, which is consistent with our miRNA-seq results [32]. It is noteworthy that hsa-miR-183-5p is predicted to bind to position 454-460 of CPEB1 3′-UTR, thus CPEB1 down-expression results in up-regulation of hsa-miR-183-5p. CPEB1 has been shown to be an important factor during oogenesis [33] and female meiotic progression [34]. Two research teams reported the CPEB1 down-expression phenotype in gastric carcinoma [35] and bone marrow mesenchymal stem cells [36]. Both groups [18,19] showed that CPEB1 silencing is caused by hyper-methylation of the promoter in experimental models. Richter reported that CPEB1 promotes TP53 mRNA polyadenylation and translation by binding to the TP53 mRNA 3′-UTR cytoplasmic polyadenylation elements [37,38]. CPEB-knockdown mice generate papillomas at a significantly faster rate than wild-type animals, suggesting the potential function of CPEB in suppressing papilloma formation. Furthermore, CPEB1 appears to provide particular protection against at least one type of induced tumor formation by inhibiting tumorigenicity [39] and decreasing angiogenic potential *in vivo* [35]. All of the existing evidence revealed that CPEB1 plays an important role against tumorigenesis. Mendezan *et al.* [23] showed that CPEB1 is a RNA-binding protein, not only regulating mRNA translation, but also mediating shortening of hundreds of mRNA 3′-UTRs, which is associated with cell proliferation and tumorigenesis. In addition, CPEB1 has been shown to cause epithelial-to-mesenchymal transition (EMT). Knockdown of CPEB1 leads to decreased protein levels of E-cadherin and beta-catenin [40], but increased expression of vimentin and Twist1. It was also shown that the motility of CPEB1-depleted cells is increased [41] and the EMT process could be promoted by p53 down-regulation [42]. Our findings suggest that CPEB1 may act as a tumor suppressor for carcinoma progression. CPEB1 is a promising target for comprehensive functional studies in EEC, and the investigation may provide insightful reference for EEC therapy development.

In this study, we have developed a method to profile the complex miRNA-mRNA interactions within EEC progressing by a multi-step strategy. As result, the EEC-related miRNA-mRNA network was constructed, and we found 61 EEC gene signatures. Pathway analysis showed that the 61 genes were enriched on many carcinoma-related pathways. In addition, among the 14 highest scoring gene signatures, we found six genes that had been well-demonstrated with endometrial carcinoma. Based on qPCR and next generation sequencing, we found that a gene signature, CPEB1, was significantly down-regulated in EEC tissues, which may have been caused by hsa-miR-183-5p up-regulation. The reliability of our prediction was based on the multi-step approach. In addition, our literature survey suggested that CPEB1 may play an important role in EEC pathogenesis by regulating the EMT/p53 pathway. There are also some limitations in this research. We still need to validate hsa-miR-183-5p by qRT-PCR in future study .mealtime, larger sample study is necessary to further confirm 10 genes expression in endometrial carcinoma tissues and matched adjacent normal endometrium. 

## 4. Materials and Methods

### 4.1. Endometrial Carcinoma-Related miRNA Collection and Target Gene Prediction

In our study, the endometrial carcinoma-related miRNAs were obtained from the following four databases: PhenomiR [43]; hmdd [44]; miRWalk [45]; and miR2disease [46]. miRNAs reported in at least two of the aforementioned databases were used for the following analysis.

Four online tools [TargetScan (http://www.targetscan.org/), miRanda (http://www.microrna.org/microrna/home.do), miRDB (http://mirdb.org/), and one experimental database starBase (http://starbase.sysu.edu.cn/)] were used for miRNA target prediction. The online tools are popular traditional miRNA target prediction software used in many publications [47,48,49]. For each miRNA, we filtered out the miRNA and target gene pairs supported by less than three methods. All of the remaining miRNA-mRNA pairs met most of the following four criteria: (a) good conservation at the 3′-UTR of mRNA (context score by Targetscan: −0.2); (b) good thermodynamic equilibrium (ΔΔ ≤ −10Kcal/mol); (c) proper binding ability/pattern between mRNA and miRNA (7mer-A/7mer/8mer); and (d) strong Clip-Seq signals in the corresponding target genes (judged by starbase). 

### 4.2. Protein-Protein Interaction (PPI) Analysis and Network Gene Scoring

The STRING database (http://string-db.org/) was utilized for gene interaction analysis. These potential targets were screened by two criteria using default settings, which have also been used [50]: (a) a score > 0.400; and (b) seven interactors were chosen (neighborhood, gene fusion, co-occurrence, co-expression, experiments, databases, and text-mining). Each gene in the resulting PPI network was scored by the number of the connected genes, and ranked by score in descending order.

### 4.3. Pathway Enrichment Analysis

The KEGG and Reactome pathway databases were used for biological pathway analysis with R/Bioconductor software. Raw p-values were adjusted using the Bonferroni method, and 0.05 was used as a cut-off.

### 4.4. Patients and Clinical Samples

Twenty-two pairs of International Federation of Gynecology and Obstetrics (FIGO) stage I EEC and adjacent non-tumorous tissues were obtained from patients (age range, 37–69 years; mean age, 55.23 ± 7.66 years) who underwent surgical excision for endometrial carcinoma at The Third Affiliated Hospital of Guangzhou Medical University from 2013–2014. The samples were collected according to the following histologic prerequisites: the carcinoma tissues had >80% tumor cells; and the matched adjacent normal endometrium had a normal mucosal structure without abnormal cells and were located at least 2 cm from the carcinoma or at least 1 cm from the carcinoma with intrauterine narrowing after extreme senile uterine contraction. The samples were snap-frozen in liquid nitrogen for 5 min after surgical resection and were then stored at −80°C until RNA extraction. Informed consent was obtained from all of the patients and the study was approved by the hospital Ethics Committee.

### 4.5. RNA Extraction

Total RNA was extracted and isolated using TRIzol (Invitrogen, Carlsbad, CA, USA) following the manufacturer’s instructions. RNA concentrations were determined with a NanoDrop apparatus (NanoDrop Technologies, Inc., and 1 ng per sample was used for the assays. RNA integrity was evaluated using the Agilent 2100 Bioanalyzer (Agilent Technologies, Palo Alto, CA, USA). The RNA integrity number (RIN) was determined using the RIN algorithm of the Agilent 2100 analyzer (Agilent Technologies, Palo Alto, CA, USA). RNAs with a RIN ≥ 7.0 and 28S/18S > 0.7 were accepted for qPCR analysis.

### 4.6. qRT-PCR

The level of mRNA expression was detected in 23 carcinoma tissues and their corresponding adjacent normal endometrium by the qRT-PCR method using an ABI Detection Kit. For RT-PCR, single-strand complementary DNA (cDNA) was synthesized from 1 μg of total RNA using the Transcriptor First Strand cDNA Synthesis Kit (Roche,) according to the manufacturer’s protocol. The primers for 15 mRNA and beta-actin (ACTB) were designed, as shown in Supplementary Materials Table S1. Real-time PCR reactions were carried out with SYBR Green PCR master. Each sample was repeated three times and the level of mRNA expression was calculated by threshold cycle (CT). The relative level of expression was calculated using 2–ΔΔCT. A median expression of each sample among all samples was chosen as a calibrator, and the housekeeping gene, beta-actin (ACTB), as an endogenous control for mRNA expression. A pairwise t-test was applied to calculate the differentially expressed level.

### 4.7. CPEB1 and hsa-miR-183-5p RPKM and Differential Calculation

The level of gene or miRNA expression detected by next generation sequencing is expressed as reads per kilo base of transcript per million mapped “reads.” In our early study, we sequenced miRNA and mRNA for FIGO stage I EEC and adjacent non-tumorous tissues to investigate the mechanisms responsible for the pathogenesis of EEC. All raw data have been deposited in the NIH Short Read Archive database (SRP045645). RPKM of CPEB1 and hsa-miR-183-5p were calculated by Cufflinks software [51], and a pairwise t-test was applied to calculate the differentially-expressed level.

## Figures and Tables

**Table 1:** Pathway analysis of top 61 protein-protein interaction (PPI) genes.

Pathway Name(ID)	Annotated Genes Quantity	Corrected *P*-value	Annotated Genes
Signal Transduction(REACT:111102)	23	1.67 × 10^−13^	CASP3|CDKN1A|CDKN1B|CHUK|COL3A1|COL4A1|E2F3|E2F5|EDN1|FRS2|GRB2|IGF1R|IRS2|NGF|NRAS|PRKAR1A|PRKCE|PTEN|SDC2|THBS1|TSC1|YAP1|YWHAB
Cell cycle(KEGG:04110)	10	1.59 × 10^−12^	CCNE2|CDC25A|CDKN1A|CDKN1B|E2F3|E2F5|ESPL1|SMC1A|YWHAB|YWHAG
Prostate cancer(KEGG:05215)	9	3.82 × 10^−12^	CCNE2|CDKN1A|CDKN1B|CHUK|E2F3|GRB2|IGF1R|NRAS|PTEN
Pathways in cancer(KEGG:05200)	12	2.70 × 10^−11^	APPL1|CASP3|CCNE2|CDKN1A|CDKN1B|CHUK|COL4A1|E2F3|GRB2|IGF1R|NRAS|PTEN
Cell Cycle(REACT:115566)	13	2.82 × 10^−10^	CCNE2|CDC25A|CDKN1A|CDKN1B|DYRK1A|E2F3|E2F5|ESPL1|NUP98|RANBP2|RRM2|SMC1A|YWHAG
Immune System(REACT:6900)	17	3.00 × 10^−10^	CDKN1A|CDKN1B|CHUK|FRS2|GRB2|IRS2|MAP3K1|NRAS|NUP98|PAK1|PRKAR1A|PRKCE|PTEN|RANBP2|TNFAIP3|WASL|YWHAB
Disease(REACT:116125)	16	4.79 × 10^−10^	CDKN1A|CDKN1B|CHUK|E2F5|FRS2|GRB2|IRS2|NRAS|NUP98|POLR2D|PRKAR1A|PRKCE|PTEN|RANBP2|SDC2|YWHAB
p53 signaling pathway(KEGG:04115)	7	2.67 × 10^−19^	CASP3|CCNE2|CDKN1A|MDM4|PTEN|RRM2|THBS1
Neurotrophin signaling pathway(KEGG:04722)	8	5.35 × 10^−19^	FRS2|GRB2|IRS2|MAP3K1|NGF|NRAS|YWHAB|YWHAG
Oocyte meiosis(KEGG:04114)	7	1.02 × 10^−7^	CCNE2|CPEB1|ESPL1|IGF1R|SMC1A|YWHAB|YWHAG
Glioma(KEGG:05214)	6	1.40 × 10^−7^	CDKN1A|E2F3|GRB2|IGF1R|NRAS|PTEN
Focal adhesion(KEGG:04510)	8	2.53 × 10^−7^	COL1A1|COL3A1|COL4A1|GRB2|IGF1R|PAK1|PTEN|THBS1
Chronic myeloid leukemia(KEGG:05220)	6	3.38 × 10^−7^	CDKN1A|CDKN1B|CHUK|E2F3|GRB2|NRAS
Small cell lung cancer(KEGG:05222)	6	8.35 × 10^−7^	CCNE2|CDKN1B|CHUK|COL4A1|E2F3|PTEN
Melanoma(KEGG:05218)	5	1.47 × 10^−5^	CDKN1A|E2F3|IGF1R|NRAS|PTEN
Extracellular matrix organization(REACT:118779)	7	3.25 × 10^−5^	CASP3|COL14A1|COL1A1|COL3A1|COL4A1|SDC2|THBS1
Developmental Biology(REACT:111045)	8	3.98 × 10^−5^	COL3A1|COL4A1|GRB2|MED8|NRAS|PAK1|WASL|YWHAB
ECM-receptor interaction(KEGG:04512)	5	4.34 × 10^−5^	COL1A1|COL3A1|COL4A1|SDC2|THBS1
MAPK signaling pathway(KEGG:04010)	7	4.94 × 10^−5^	CASP3|CHUK|GRB2|MAP3K1|NGF|NRAS|PAK1
ErbB signaling pathway(KEGG:04012)	5	5.14 × 10^−5^	CDKN1A|CDKN1B|GRB2|NRAS|PAK1
Bladder cancer(KEGG:05219)	4	8.68 × 10^−5^	CDKN1A|E2F3|NRAS|THBS1
Cellular responses to stress(REACT:120956)	6	2.53 × 10^−4^	CBX4|CCNE2|CDKN1A|CDKN1B|E2F3|HMGA2
Insulin signaling pathway(KEGG:04910)	5	5.15 × 10^−4^	GRB2|IRS2|NRAS|PRKAR1A|TSC1
Protein digestion and absorption(KEGG:04974)	4	0.00123	COL14A1|COL1A1|COL3A1|COL4A1
Apoptosis(KEGG:04210)	4	0.00179	CASP3|CHUK|NGF|PRKAR1A
Chemokine signaling pathway(KEGG:04062)	5	0.00201	CHUK|GRB2|NRAS|PAK1|WASL
Fc gamma R-mediated phagocytosis(KEGG:04666)	4	0.00262	CFL2|PAK1|PRKCE|WASL
Amoebiasis(KEGG:05146)	4	0.0037	CASP3|COL1A1|COL3A1|COL4A1
T cell receptor signaling pathway(KEGG:04660)	4	0.00412	CHUK|GRB2|NRAS|PAK1
Binding and Uptake of Ligands by Scavenger Receptors(REACT:160300)	3	0.00499	COL1A1|COL3A1|COL4A1
Axon guidance(KEGG:04360)	4	0.00865	CFL2|EFNB2|NRAS|PAK1
Extracellular matrix organization(REACT:195275)	2	0.00935	COL1A1|SDC2
Hepatitis C(KEGG:05160)	4	0.00998	CDKN1A|CHUK|GRB2|NRAS
Measles(KEGG:05162)	4	0.00998	CCNE2|CDKN1B|CHUK|TNFAIP3
Natural killer cell mediated cytotoxicity(KEGG:04650)	4	0.01177	CASP3|GRB2|NRAS|PAK1
Endometrial cancer(KEGG:05213)	3	0.01232	GRB2|NRAS|PTEN
Non-small cell lung cancer(KEGG:05223)	3	0.01232	E2F3|GRB2|NRAS
Acute myeloid leukemia(KEGG:05221)	3	0.01448	CHUK|GRB2|NRAS
Apoptosis(REACT:578)	4	0.01603	APPL1|CASP3|YWHAB|YWHAG
Hemostasis(REACT:604)	6	0.01944	COL1A1|GRB2|NRAS|PRKAR1A|PRKCE|THBS1
Epithelial cell signaling in Helicobacter pylori infection(KEGG:05120)	3	0.02448	CASP3|CHUK|PAK1
Gene Expression(REACT:71)	8	0.02501	E2F5|IGF2BP1|MED8|NR3C1|POLR2D|SMC1A|YAP1|YWHAB
Renal cell carcinoma(KEGG:05211)	3	0.02783	GRB2|NRAS|PAK1
B cell receptor signaling pathway(KEGG:04662)	3	0.03815	CHUK|GRB2|NRAS
Fc epsilon RI signaling pathway(KEGG:04664)	3	0.04732	GRB2|NRAS|PRKCE

**Table 2:** Literature survey of initial endometrial carcinoma-related genes.

Gene Symbol	#of PPIs Neighbor	Relationship with Endometrial Carcinoma	Reference
PTEN	25	PTEN mutation is commonly found in endometrial carcinoma.PTEN expression is a diagnostic marker and poor prognostic factor.	[12,13,14,15]
CDKN1A(p21)	15	CDKN1A is a significantly mutated gene in endometrial carcinoma.Genetic polymorphisms and susceptibility to endometrial carcinoma	[16,17,18,19]
NRAS	15	NRAS is a significantly mutated gene in endometrial carcinoma.	[20]
CDC25A	15	Thought to be a oncogene in endometrial carcinoma	[21,22]
CDKN1B(p27Kip1)	14	CDKN1A has genetic polymorphisms and susceptibility to endometrial carcinomaCDKN1A has therapeutic potential for endometrial carcinoma	[19,23,24,25]
IGF1R	14	Potential therapy target of endometrioid adenocarcinoma	[26,27,28]

**Table 3:** qRT-PCR validation of 10 genes with 6 pairs of endometrial carcinoma tissues and matched adjacent normal endometrium.

ID	Genes	Matched Adjacent Endometrium	Endometria Carcinoma Tissues (Mean ± SD)	*p* Value
1	CDC25A	1	27.322 ± 19.981	0.04
2	IGF1R	1	4.742 ± 3.501	0.04
3	CPEB1	1	0.417 ± 0.574	0.05
4	GRB2	1	3.962 ± 5.03	0.17
5	ACTA1	1	4.135 ± 4.376	0.14
6	IRS2	1	2.771 ± 2.721	0.17
7	CASP3	1	2.802 ± 3.935	0.27
8	NGF	1	4.546 ± 9.295	0.39
9	YWHAB	1	1.082 ± 0.618	0.73
10	SDC2	1	1.08 ± 1.134	0.85

## Supplementary Materials

The following are available online at http://www.mdpi.com/2073-4425/7/6/26/s1.

## Abbreviations

The following abbreviations are used in this manuscript:

EEC	endometrioid endometrial carcinoma
PPI	protein-protein interaction
