# Supplementary material for: A Multi-Step miRNA-mRNA Regulatory Network Construction Approach Identifies Gene Signatures Associated with Endometrioid Endometrial Carcinoma
Source: Genes (Basel). 2016 Jun 2;7(6):26. doi: 10.3390/genes7060026 (PMC4929425; doi:10.3390/genes7060026)
Supplement: Supplementary file 1 [file genes-07-00026-s001.pdf]

Supplementary Materials: A multi-step miRNA-mRNA Regulatory Network Construction Approach Identifies Gene Signatures Associated with Endometrioid Endometrial Carcinoma

Hanzhen Xiong, QiulianLi, Ruichao Chen, Shaoyan Liu, Qiongyan Lin, Zhongtang Xiong, Qingping Jiang and Linlang Guo

Table S1. Sequence of Primers for RT-PCR.

Gene	Name	mRNA no.	F-primer	R-primer	Product Size
GRB2	growth factor receptor-bound protein 2	NM_002086.4	ACGGCTTCATTCCCAAGAACT	TCCAAACTTGACAGAGAGGGAGA	173
PTEN	phosphatase and tensin homolog	NM_000314.4	ACAATCTTTGTGCTGAAAGACATTA	CACCAGTTCGTCCCTTTCCA	195
NGF	nerve growth factor	NM_002506.2	GGGAGCGCAGCGAGTTTTG	TTAAACAGCCTGGGGTCCAC	381
CDC25A	Cell division cycle 25A	NM_001789.2	CCCTACCTCAGAAGCTGTTGG	GCAGAGTTCTGCCTCTGTGT	217
NRAS	neuroblastoma RAS viral (v-ras) oncogene homolog	NM_002524.4	GCTTGAGGTTCTTGCTGGTGT	CGCCTGTCCTCATGTATTGGT	250
CDKN1A	cyclin-dependent kinase inhibitor 1A (p21, Cip1)	NM_000389.4	GCCGAAGTCAGTTCCTTGTG	TCGAAGTTCCATCGCTCACG	230
CASP3	caspase 3, apoptosis-related cysteine peptidase	NM_004346.3	GCCGTGAGGAGTTAGCGAG	CGAGATGTCATTCCAGTGCT	293
IGF1R	insulin-like growth factor 1 receptor	NM_000875.4	TCTATGTCCAGGCCAAAACAGG	GTGATCTTCTCCCGAGCCAC	235
IRS2	insulin receptor substrate 2	NM_003749.2	TCTCAGGAAAAGCAGCGAGG	TCACGTCGATGGCGATGTAG	233
CDKN1B	cyclin-dependent kinase inhibitor 1B (p27, Kip1)	NM_004064.4	ACCCGGGAGAAAGATGTCAAA	TGCCCTCTAGGGGTTTGTGA	230
CPEB1	cytoplasmic polyadenylation element binding protein 1	NM_001079533.1	GCCTAAAGGGTATGTGTATCTGG	CATGCAGAGCACCGACAAAC	245
ACTA1	actin, alpha 1, skeletal muscle	NM_001100.3	AATGTGCGACGAAGACGAGA	ATGATGCCGTGCTCGATAGG	234
SDC2	syndecan 2	NM_002998.3	GCCGGTCCCTGGGGAATATG	TCTACATCCTCATCAGCTCCCGA	208
CCNE2	cyclin E2	NM_057749.2	GTAGCTGGTCTGGCGAGGTT	TAATGCAAGGACTGATCCCCC	252
YWHAB	tyrosine 3-monooxygenase/tryptophan 5-monooxygenase activation protein, beta	NM_003404.4	GGCCGCTACAAAAAGTGCAT	CATGCCCCTGTTCTGTGACT	187
ACTB	Beta-actin	NM_001101.3	CTCACCATGGATGATGATATCGC	AGGAATCCTTCTGACCCATGC	163

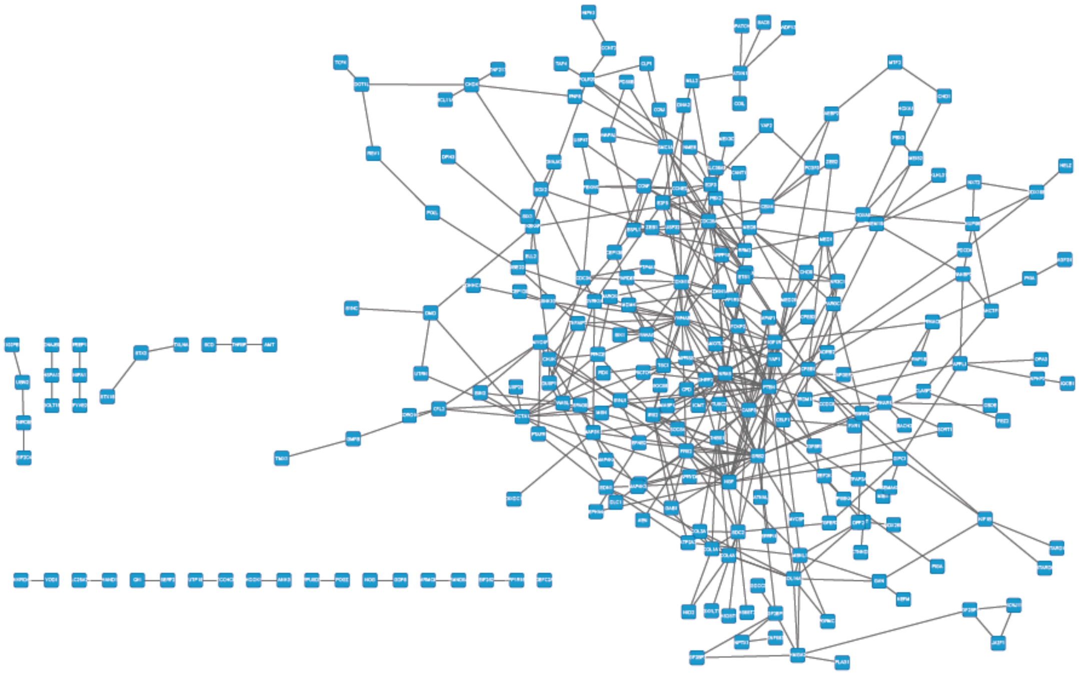

Figure S1. 61 of the potential targets possess a high score (≥5).

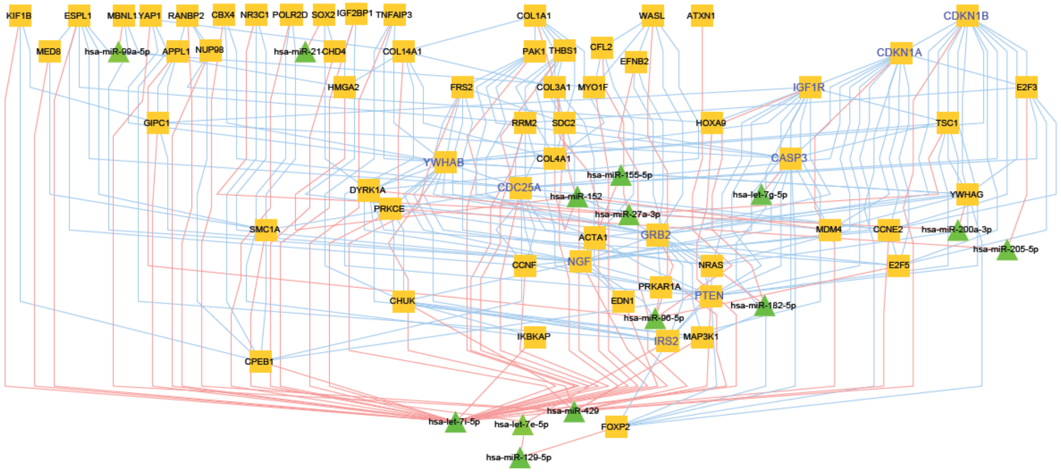

Figure S2. The interactions of 61 genes and the relationships with miRNAs.
